# Overweight and Obesity among Low-Income Muslim Uyghur Women in Far Western China: Correlations of Body Mass Index with Blood Lipids and Implications in Preventive Public Health

**DOI:** 10.1371/journal.pone.0090262

**Published:** 2014-02-28

**Authors:** Li Cong, Jin Qiong Zhan, Lan Yang, Wei Zhang, Shu Gang Li, Cheng Chen, Hong Yan Zhang, Zhi Ping Ma, Xiao Ling Hao, Dilixia Simayi, Lin Tao, Jin Zhao, A. Amanguli, Meiliguli Mohemaiti, Ming Xia Jing, Wei Wang, Abudukeyoumu Saimaiti, Xiao Guang Zou, Yan Gu, Li Li, Ying Hong Wang, Feng Li, Wen Jie Zhang

**Affiliations:** 1 Department of Pathology, Shihezi University School of Medicine, Shihezi, Xinjiang, China; 2 The Key Laboratories for Xinjiang Endemic and Ethnic Diseases, Shihezi University, Shihezi, Xinjiang, China; 3 Department of Obstetrics and Gynecology, the First Affiliated University Hospital, Shihezi University School of Medicine, Shihezi, Xinjiang, China; 4 Faculty of Preventive Medicine, Shihezi University School of Medicine, Shihezi, Xinjiang, China; 5 Institute of Humanities, Shihezi University School of Medicine, Shihezi, Xinjiang, China; 6 Department of Oncology, the First People's Hospital of Suqian, Suqian, Jiangsu, China; 7 Department of Laboratory Medicine, JiangXi Mental Health Center, Nanchang, Jiangxi, China; 8 Department of Immunization, Xi'an Center for Disease Control and Prevention, Xi'an, Shanxi, China; 9 Kashi Prefecture First People's Hospital, Kashi Prefecture Health Bureau, Kashi, Xinjiang, China; 10 Department of Obstetrics and Gynecology, Institute of Surgery Research, Daping Hospital, the Third Military Medical University, Chongqing, China; University of Sao Paulo, Brazil

## Abstract

**Background:**

The pandemic of obesity is a global public health concern. Most studies on obesity are skewed toward high-income and urban settings and few covers low-income populations. This study focused on the prevalence of overweight and obesity and their correlations with blood lipids/metabolites/enzymes (bio-indicators) in a rural community typical of low-income in remote western China.

**Methods:**

This study was performed in a Muslim ethnic Uyghur rural community in Kashi Prefecture of Xinjiang, about 4,407 km (2,739 miles) away from Beijing. Body mass index (BMI) and major blood bio-indicators (25 total items) were measured and demographic information was collected from 1,733 eligible healthy women aged 21 to 71 yrs, of whom 1,452 had complete data for analysis. More than 92% of the women lived on US$1.00/day or less. According to the Chinese criteria, overweight and obesity were defined as BMI at 24 to <28 kg/m^2^ and at ≥28 kg/m^2^, respectively.

**Results:**

The average BMI among these low-income women was 24.0±4.0 (95% CI, 17.5–33.7) kg/m^2^. The prevalence of obesity and overweight was high at 15.1% and 28.9%, respectively. Among 25 bio-indicators, BMI correlated positively with the levels of 11 bio-indicators including triglycerides (TG), low-density lipoprotein cholesterol (LDL-C), total cholesterol (TCHOL), glucose (GLU), and uric acid (UA); but negatively with the levels of 5 bio-indicators including high-density lipoprotein cholesterol (HDL-C) and apolipoprotein A/B (APO A/B).

**Conclusions:**

This is the first investigation reporting overweight and obesity being common in low-income Muslim Uyghur women, whose BMI correlates with several important blood bio-indicators which are risk factors for diabetes and cardiovascular diseases. These findings may help make preventive public health policies in Uyghur communities. To prevent diabetes and cardiovascular diseases in low-income settings, we therefore propose a cost-effective, two-step strategy first to screen for obesity and then to screen persons with obesity for diabetes and cardiovascular diseases.

## Introduction

Obesity has become a global pandemic affecting 200 million men and nearly 300 million women worldwide and posting great public health threats to all nations and all races [1]. Obesity causes a myriad of health problems from aspects of ill health, functional impairment and reduced quality of life, to serious diseases and greater mortality [2]. Obesity has recently been identified as a disease by American Medical Association [3], a major leap forward in diagnosis, treatment and prevention of obesity and related diseases as well as mortality.

In China, an analysis has coincided obesity with economic growth and family income [4]. Indeed, the fast economic growth in the past three decades in China has dramatically improved access to high energy foods and prompted significant lifestyle changes manifested by overconsumption of dietary fat and sweetened soft-drinks, much increased binge eating behavior [5] and much decreased physical activities, all of which may have contributed to an increasing prevalence of overweight and obesity [6]–[8]. For example, A report from China has found that overweight or obesity has reached 25.6% in the urban, and 17.3% in the rural populations, respectively in 2000, more than doubled as compared with the rates in 1989 (12.2% and 7.7%, respectively) [9]. At a national level, the epidemic trends of overweight and obesity have progressed to an alarming point in the Chinese population, which has clearly become a major public health issue [7], [10].

Thus far, most studies on obesity have been performed in urban and/or high-income settings and few covers low-income populations. In a screening program for cervical cancer in a Muslim Uyghur minority township typical of low-income setting in Kashi Prefecture, Xinjiang, located in remote western China, about 4,407 km (2,739 miles) away from Beijing [11], we have simultaneously performed a rural community health investigation on the prevalence of overweight and obesity in low-income women and tested their major blood lipids, metabolites, and enzymes (bio-indicators). We report for the first time an investigation in low-income Muslim Uyghur women and show that (1) overweight and obesity are common, and (2) Body mass index (BMI) correlates with several blood bio-indicators suggested in type 2 diabetes and cardiovascular diseases. These findings may have important implications in preventive public health policies in low-income Muslim Uyghur rural communities.

## Methods

### Ethics Statement

The Institutional Ethics Review Board (IERB) at the First Affiliated Hospital of Shihezi University School of Medicine approved the study (IERB No. SHZ2008LL01). Standard university hospital guidelines including informed consent, voluntary participation, confidentiality, and anonymity were followed. All participants gave written informed consent before the study began.

### Settings and Participants

The investigation was part of a screening program for cervical cancer performed from November to December 2010 in Jiangbazi Township of Jiashi (Payzawat in Uyghur language) County where approximately 98% of the population are minority Muslim Uyghurs. Most of rural Uyghur residents still live in traditional lifestyle with little changes after 3 decades of China's economic reform. Jiashi County is one of the poorest counties in China located in Kashi (Kashgar) Prefecture, Xinjiang Uyghur Autonomous Region in remote western China. In a local primary hospital, demographic information and blood were collected from participants.

### Selection Criteria

Of 1,733 women who were recruited consecutively as they presented for cervical cancer screening, 1,452 were regarded healthy and included in the final analyses because: (1) they reported no chronic diseases including diabetes and cardiovascular diseases; (2) They had both normal Pap tests and visual inspection with acidic acid (VIA) or not been diagnosed as having cervical intraepithelial neoplasia (CIN, grade 1, 2, or 3) or cervical cancer; and (3) they provided demographic information and had blood taken for biochemistry tests.

### Body Mass Index (BMI) and Categorization

Participants, wearing light cloths with shoes off, were measured for body weight to the nearest 0.1 kg, and height to the nearest 0.1 cm using a balance-beam scale with a ruler [12], [13]. BMI was calculated as weight (kilogram or kg) divided by height (meter or m) squared (kg/m^2^). According to the Chinese Working Group on Obesity [14]–[16], underweight was defined as BMI≤18.5 kg/m^2^, normal weight as BMI>18.5 to 23.9 kg/m^2^, overweight as BMI≥24 to 27.9 kg/m^2^, and obesity as BMI≥28 kg/m^2^, respectively. The WHO definitions for obesity [1] were also used for comparative analyses.

### Blood Biochemistry Tests

Blood was drawn from the antecubital vein after fasting overnight and plasma was obtained by centrifugation at 3,000 rpm for 30 minutes at 4°C and stored at −70°C until test. Employing Olympus AV2700 Biochemical Automatic Analyzer (Olympus, Japan), we tested a total of 25 blood biochemicals, lipids, enzymes and metabolites (referred to as bio-indicators). Plasma glucose and lipids were measured using a modified hexokinase enzymatic method while levels of total cholesterol (TC), high-density lipoprotein (HDL), cholesterol and triglycerides (TG) and other biochemicals were analyzed enzymatically in the Biochemistry Laboratory, the First University-Affiliated Hospital of Shihezi University School of Medicine.

### Statistical Analysis

A databank was created that included all participants' demographic information, BMI, and biochemistry test results using EpiData software (EpiData Association, Odense, Denmark, http://www.epidata.dk/). Data were then analyzed using SPSS (Statistical Program for Social Sciences, version 17.0, 2008), which allowed for complex survey designs used for calculating estimates. All data were examined for distributions and outliers. Outliers were identified and recoded to the 95th percentile of the distribution. Descriptive statistics were computed for all variables, including means for continuous variables, standard deviation (SD) of the mean, frequencies, and standard errors (SEs) for categorical variables. Spearman rank correlation was used to analyze the relationship between BMI and biochemical indicators and ANOVA (analysis of variance) was used to analyze differences across groups and *post hoc* comparisons were also performed between certain groups (Table 1). All *P* values are 2-sided, and statistical significance was defined as *P*<0.05.

**Table 1:** BMI-based distributions of age, income, BMI and the levels of blood bio-indicators among four BMI categories.

Variables (unit)	BMI-UW (<18.5)	BMI-NW (18.5–23.9)	BMI-OW (24–27.9)	BMI-OB (≥28)	Total or average
No. of individuals	77	738	422	219	1,452
% of total	5.2	50.8	28.9	15.1	100
Age (yrs)	34.8±0.8	38.8±8.4	39.6±8.3	39.8±8.3	39.3±8.5
BMI (kg/m^2^)***	17.4±0.8	21.6±1.5	25.8±1.2	30.9±2.6	24.0±4.0
Per capita income/day (US$)^#^	0.38±0.41^#^	0.69±1.04	0.56±0.88	0.67±0.95	0.59±0.96
Uric acid (mg/L)***	156±56	173±53	178±51	183±49	175±52
High-density lipoprotein cholesterol (mmol/L)***	1.26±0.30	1.21±0.27	1.19±0.27	1.11±0.26^a^	1.19±0.27
Flavin mononucleotide* (mmol/L)	240±66	235±32	232±25	229±31	234±33
Alkaline phosphatase (U/L)***	75±24	72±24	77±34	80±26	75±28
Triglycerides (g/L)**	0.93±0.53	1.07±0.83	1.21±0.75	1.30±0.79^a^	1.14±0.96
Low-density lipoprotein cholesterol (mmol/L)*	2.33±0.83	2.39±0.76	2.47±0.76^b^	2.44±0.71^b^	2.42±0.76
Glucose (mg/L)*	5.24±0.81	5.38±1.26	5.47±0.92	5.50±0.90^a^	5.44±1.26
Calcium (mg/L)*	2.43±0.12	2.39±0.13	2.39±0.13	2.38±0.12	2.39±0.13
Alanine aminotransferase (IU/L)	17±15	16±24	16±13	16±8	16±19
Albumin/Globulin (mmol/L)	1.7±0.3	1.7±0.3	1.69±0.3	1.7±0.3	1.7±0.3
Apolipoprotein B (g/L)	0.95±0.25	0.96±0.24	0.98±0.23	0.97±0.23	0.97±0.23
Apolipoprotein A/B	1.6±0.5	1.6±0.5	1.6±0.4	1.6±0.6	1.6±0.5
Hydroxybutyrate dehydrogenas (IU/L)	130±27	138±38	138±30	139±26	138±34
Glutamyl endopeptidase (mmol/L)	13±10	13±17	14±12	15±17	14±15
Lactate dehydrogenase (IU/L)	166±31	176±53	177±38	178±34	176±46
Total cholesterol (mg/L)	4.15±1.01	4.14±1.01	4.28±1.05	4.26±0.95	4.20±1.01

Note: BMI categories are based on cutoff values for Chinese [14]–[16]: UW = underweight, NW = normal weight, OW = overweight, OB = obesity. Values are mean ± standard error. ANOVA (analysis of variance) is used to analyze differences among BMI-based categories: * = *P*<0.05, ** = *P*<0.01, and *** = *P*<0.001. Comparisons between groups: ^a^ = OB is significantly different from OW, NW or UW (*P*<0.05, *P*<0.005 or *P*<0.001, respectively); ^b^ = OB or OW is significantly different from NW or UW (*P*<0.05 and *P*<0.05, respectively). ^#^US$1 = CN¥6.623 (31 December 2010, Bank of China), and per capita income/day in BMI-UW category is lower than that in three other BMI categories, respectively (*P*<0.05). Per capita income of US$0.50 per day (US$181 per year) was the Chinese rural poverty line in 2010 and before.

## Results

### Participants Are Typical Low-income Muslim Uyghur Women

According to the selection criteria, we were able to retain 1,452 eligible healthy Uyghur women with adequate data from 1,733 participants (Table 1). Among these low-income women, more than 92% of them lived on or below US$1.00 per day and as many as 29% of them were illiterate (no school at all).

### Overweight and Obesity Are Common in This Low-income Population

The 1,452 female participants' ages spanned from 21 to 71 years with an average of 39.3±8.5 years. As shown in Table 1, this population had an average BMI of 24.0±4.0 kg/m^2^ (95% CI, 17.5–33.7). According to the Chinese Working Group on Obesity [14], [15], the prevalence of obesity (BMI≥28 kg/m^2^) was 15.1% and, of overweight (BMI≥24 to 27.9 kg/m^2^) reached 28.9% (Table 1). However, if adopting the WHO reassessed body mass index for Asian populations [19], the prevalence of obesity (BMI≥25 kg/m^2^) in this population would be 36.3%, more than doubling 15.1% as shown above and, the prevalence of overweight would be lowered from 28.9% to 18.1% due to a narrower BMI interval (BMI = 23–24.9 kg/m^2^) [19]. We used the Chinese BMI definitions in our final analyses.

### BMI-based Distributions of Age, Income and Blood Biochemical Indicators

Based on four BMI definitions as shown in Table 1, 5.2% of the women were underweight (BMI≤18.5 kg/m^2^); over a half (50.8%) of them was normal weight (BMI>18.5 to 23.9 kg/m^2^); 28.9% of them were overweight (BMI≥24 to 27.9 kg/m^2^); and 15.1% of whom were regarded as obese (BMI≥28 kg/m^2^). Age did not appear to correlate with BMI categories although the age in underweight women was younger. However, per capita income per day among underweight women was lower than that among women in three other BMI categories, respectively (*P*<0.05).

**Figure 1:**
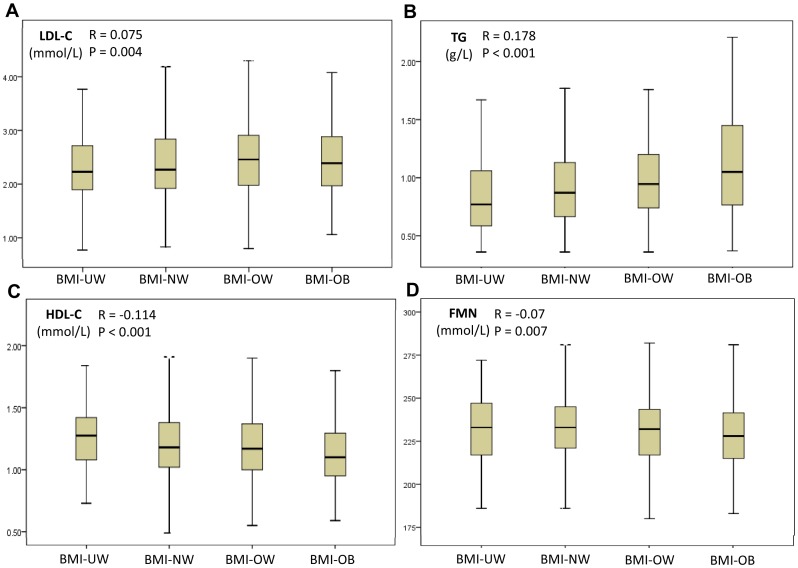
BMI is positively correlated with LDL-C (A) and TG (B), but negatively correlated with HDL-C (C) and FMN (D). Presented are four examples of such correlations as shown in Table 2 using Pearson analyses with R values and *P* values. BMI categories are defined as in Table 1 and plasma levels (with units shown) of lipids are presented as box and whisker plots as median (thicker bar in the box), the 25th (lower edge of the box), and 75th (upper edge of the box) percentiles. LDL-C, low-density lipoprotein cholesterol; TG, triglycerides; HDL-C, high-density lipoprotein cholesterol; FMN, flavin mononucleotide.

It was interesting to note that levels of several blood lipids and metabolites commonly tested in clinical laboratories were unambiguously differentiated according to 4 BMI categories, suggesting the usefulness of the BMI definitions [20]. And, these common blood lipids and metabolites showed an upward trend of increasing levels along with increasing BMI, including triglycerides (TG), glucose (GLU), alkaline phosphatase (ALP) and uric acid (UA), which were higher in overweight and obese women relative to normal weight persons by two-group comparisons (Table 1), suggesting their potential implications clinically. Reciprocally, however, a downward trend had been noticed for high-density lipoprotein cholesterol (HDL-C), flavin mononucleotide (FMN) and calcium (Ca) whose levels were decreased as BMI was increased (Table 1), which showed lower levels in overweight and obese persons compared with normal weight persons. Examples of up- (TG and LDL-C) and down-trend (HDL-C and FMN) bio-indicators were presented in Figure 1.

### Correlations of BMI with Major Blood Biochemical Indicators

As both BMI and major blood lipids are associated with cardiovascular diseases, correlations of BMI with these blood lipids would be expected. Indeed as shown in Table 2, when Pearson analyses were used to characterize the correlations between BMI and blood biochemical indicators, 11 of 16 lipids, enzymes, and metabolites showed significant positive correlations, of which many have been risk factors or indicators for cardiovascular diseases such as triglycerides, low-density lipoprotein cholesterol, and total cholesterol (TCHOL), or for type 2 diabetes such as glucose. The remaining 5 bio-indicators (lower part in Table 2), however, exhibited negative correlations with BMI. Four typical such correlations (2 positives and 2 negatives) were presented in Figure 1 using box and whisker plots. The findings described above were in keeping with those observations of relevant up- or down-trending bio-indicators in relation to BMI categories (Table 1).

**Table 2:** Correlations of BMI with blood lipids, enzymes and metabolites among 1,452 low-income women in a rural community of Kashi Prefecture, Xinjiang, China.

Variables	Mean±SD	95% CI	Pearson R value	*P* value
BMI (kg/m^2^)	24.0±4.0	(17.5, 33.7)	/	/
Triglycerides (g/L)	1.14±0.96	(0.37, 2.93)	0.178	<0.001
Glutamyl endopeptidase (mmol/L)	14±15	(4, 37)	0.147	<0.001
Uric acid (mg/L)	175±52	(96, 293)	0.128	<0.001
Alkaline phosphatase (U/L)	75±28	(39, 133)	0.094	<0.001
Lactate dehydrogenase (IU/L)	176±46	(110, 263)	0.094	<0.001
Total cholesterol (mg/L)	4.20±1.01	(2.44, 6.28)	0.081	0.002
Alanine aminotransferase (IU/L)	16±19	(7, 36)	0.080	0.002
Hydroxybutyrate dehydrogenase (IU/L)	138±34	(93, 205)	0.077	0.003
Low-density lipoprotein cholesterol (mmol/L)	2.42±0.76	(1.13, 4.04)	0.075	0.004
Glucose (mg/L)	5.44±1.26	(4.08, 7.29)	0.075	0.004
Apolipoprotein B (g/L)	0.97±0.23	(0.52, 1.45)	0.057	0.031
Calcium (mg/L)	2.39±0.13	(2.16, 2.63)	−0.057	0.031
Albumin/Globulin (mmol/L)	1.7±0.3	(1.2, 2.3)	−0.060	0.026
Flavin mononucleotide (mmol/L)	234±33	(193, 279)	−0.070	0.007
Apolipoprotein A/B ratio	1.6±0.5	(0.5, 1.5)	−0.088	0.001
High-density lipoprotein cholesterol (mmol/L)	1.19±0.27	(0.73, 1.77)	−0.114	<0.001

Note: Mean±SD with 95% confidence intervals (CI) is used to describe BMI and blood biochemical indicators. Pearson R values with *P* values are the description of correlations between BMI and biochemical indicators listed.

## Discussion and Conclusions

Thus far, studies on obesity are largely concentrated in high-income and/or urban populations due to higher rates of overweight and/or obese individuals in these populations. Attentions to obesity in low-income populations are largely neglected due to reasons including, but not limited to, lower rates of obesity, low public health priority, lack of local expertise, poor transportation and dispersed residency among many others. This disparity results in limited information for public health policy-making in low-income settings for awareness education and preventive measures relevant to obesity. Here we report for the first time the reference ranges of 4 BMI categories and the prevalence of overweight and obesity (Table 1) in a typical low-income western township where approximately 98% of the population are minority Muslim Uyghurs. Among these low-income women, more than 92% of them lived on US$1.00 per day or less, a sharp contrast to the national average of 15.9% of people living on <US$1.00 per day in 2005 [11], [17]. Furthermore, the illiteracy (no school) was as high as 29% among these women, a rate dramatically higher than the national average of 4.1% in 2010 [11], [18].

By adopting the BMI definitions established by the Chinese Working Group on Obesity [20], we have found both overweight and obesity to be common with 28.9% for overweight and 15.1% for obesity, respectively, among low-income women (Table 1). As shown in Table 3, this obesity rate (15.1%) in rural low-income Uyghur women is significantly lower than urban Uyghur women who show a striking obesity rate of 56.9% in a large city (Urumqi, Xinjiang), but is more than doubled as compared with the obesity rate of 7.1% in Han ethic populations [21]. Even by adopting the WHO criteria for obesity [1], we have observed high enough rates for overweight (28.3%) and obesity (8%) among these low-income Uyghur women, whose overweight and obesity rates are higher than female Africans of a rural low-income setting (17.6% for overweight and 5.2% for obesity) [22] and, much higher than urban/rural female Han Chinese and urban female Japanese (Table 3). It should not be ignored that per capita income, by which living standard is based, plays a role in determining BMI-based underweight individuals in this low-income population (Table 1).

**Table 3:** A comparison of overweight and obesity according to BMI categorization among females from selected ethnic groups within China and in the world.

		n		BMI Category (%)				
Ethnicity	Residency		Age (y)	UW	NW	OW	OB	Reference
Uyghur	Jiashi rural, China	1,452	21–71	5.2	50.8	28.9	15.1	this study*
Uyghur	Urumqi urban, China	260	40–74	/	/	26.5	56.9	[35]*
Han	Urban & rural, China	272,023	≥18	/	/	22.8	7.1	[36]*
Uyghur	Jiashi rural, China	1,452	21–71	5.2	58.5	28.3	8.0	this study^#^
Han	Urban & rural, China	272,023	≥18	/	/	18.9	2.9	[36] ^#^
Turkish	Urban & rural, Turkey	1,942	20–85	5.9	43.9	33.6	16.6	[37] ^#^
Japanese	Urban, Japan	7,153	≥20	44.2	24.6	28.0	3.2	[38] ^#^
Black	Rural, West Africa	199	30–50	/	/	17.6	5.2	[39] ^#^
Black	Urban, South Africa	5,495	≥18	/	/	32.0	36.0	[40] ^#^
Black	Norway	208	≥25	/	/	OW+OB = 66		[41] ^#^
Black	Non-Hispanic, USA	2,490	≥20	/	/	29.4	36.7	[42] ^#^
White	Hispanic, USA	2,128	≥20	/	/	32.5	33.3	[42] ^#^
White	Non-Hispanic, USA	3,755	≥20	/	/	24.8	22.7	[42] ^#^

Note: UW = underweight, NW = normal weight, OW = overweight, OB = obesity. / = Data not available. * = Using BMI cutoff values recommended for Chinese [14]–[16], ^#^ = Using BMI cutoff values recommended by WHO (overweight if BMI≥25–29.9 kg/m^2^ and obesity if BMI≥30 kg/m^2^). Han = Chinese ethnic Han majority. See Table 1 for more footnotes.

Obesity is a disease with complex trait which results from a variety of genetic, behavioral, and environmental determinants [8]. Rural Muslim Uyghurs maintain an agricultural mode of subsistence with wheat, sheep milk and lamb as their major foods. Given the low-income environment these Uyghurs live in, rare high energy foods and disordered eating behaviors seem unlikely [5], although these factors are in need of further study. It is more likely that dietary composition and genetics may play important roles in Uyghur obesity. Molecular studies have demonstrated that Uyghurs have a mixture of 60% European ancestry and 40% East Asian ancestry [23], which may explain, at least in part, the genetic influence on the studied Uyghurs whose obesity rate is higher than other Asians (Han Chinese and Japanese) but lower than Europeans (Whites and Hispanics) (Table 3).

We have further analyzed the relationship between BMI categories and 16 commonly tested blood biochemical indicators in this low-income female population. It is interesting to note that, along with the increasing BMI, the levels of 11 bio-indicators show a trend of linear increase, which include TG, LDL-C, LDH, TCHOL, HBDH, GLU, ALT, UA, ALP and GGT, and reciprocally, a trend of linear decrease is observed for the levels of HDL-C, APO A/B, Ca, FMN, A/G, and BUN (Table 1), in keeping with the Pearson correlation analyses (Table 2, Figure 1). Moreover, compared with the women with a normal BMI, the overweight and obesity women show the significantly higher levels of TG, GLU, ALP, UA, and HDL-C (overweight vs. normal weight) but the significantly lower levels of HDL-C, FMN and Ca, respectively (Table 1). For many years, associations have been revealed between BMI and triglycerides and between BMI and lipid profiles, which are in agreement with our observations as shown above.

Obesity is a disease that is associated with or related to many other diseases, such as metabolic syndromes and cardiovascular diseases. For example, obese children and adolescents appear to have a higher risk of developing metabolic syndrome than non-obese children [24]. In comparison with normal weight individuals, overweight individuals have 4.7 times higher probability of suffering from metabolic syndromes, and the probability increases to 30 times higher in obese individuals [25]. Both lipid profile and BMI have been shown to be important predictors for hypertension, diabetes, and cardiovascular diseases [26], [27], and clinical data indicate that elevated levels of total cholesterol, triglycerides and low-density lipoprotein are risk factors for cardiovascular events [28], [29]. Obesity is consistently associated with unhealthy lipid profiles characterized by high triglycerides and LDL but low HDL [18], in keeping with our findings as shown in Table 1. Obesity has been reported to be a major risk factor for diabetes mellitus in Iraqi diabetic premenopausal Muslim women [30]. As compared with Han Chinese, Uyghur women exhibit much higher prevalence of obesity (Table 3) which correlates with higher levels of blood glucose and lipids than normal weight (Table 1). Our observations coincide with the clinical findings that Muslim Uyghurs with type 2 diabetes show higher rates of obesity, hyperlipidemia, hypertension, and coronary heart disease than Han Chinese diabetic patients living in the same environment [31], [32].

It has been noted that relative risks of mortality are associated with increased BMI indices and, relative to normal weight population, obese population has much higher excess deaths [33]. Furthermore, in the cancer front of obesity-related risks, obese women may be more vulnerable to cancer occurrence and more likely to miss routine cancer screening, placing them at a greater risk for delayed cancer diagnosis and poor prognosis [34], a significant concern in low-income rural areas where access to healthcare is limited such as the rural Uyghur communities we performed cervical cancer screening [11].

In summary, this is the first time report of a community health investigation in a typical low-income Muslim Uyghur setting where more than 92% of the participating women live on US$1.00 or less per day and 29% of them are illiterate, both dramatically higher than the national averages [11], [18]. We have shown that (1) BMI-defined overweight (28.9%) and obesity (15.1%) are common among these low-income Uyghur women; (2) The established reference ranges for four BMI categories in this population appear to differentiate blood levels of major blood lipids, glucose and metabolites; and (3) BMI correlates with several blood bio-indicators that have been suggested in type 2 diabetes and cardiovascular diseases. Uyghur ethnic group is a minority in China but there are more than 10 million Uyghurs in Xinjiang Uyghur Autonomous Region, representing 46.2% of the region's total population [18]. In Kashi (Kashgar) Prefecture, most Uyghurs reside in low-income rural communities where public health resources are limited but chronic diseases such as type 2 diabetes and cardiovascular diseases are common [31]. As BMI correlates with blood glucose and lipids which are associated with diabetes and cardiovascular diseases, one cost-effective public health policy in preventing diabetes and/or cardiovascular diseases in low-income settings would be to offer a two-step screening strategy: first to screen for obesity using the least costive BMI index and, then to prioritize persons with obesity using higher costive but subsidized screening procedures for diabetes and cardiovascular diseases. This approach, similar in principle to our proposed semi-mandatory HPV vaccination strategy for preventing cervical cancer in low-income settings [11], may prove to be realistic and can yield significant public health benefits for low-income individuals who cannot afford sophisticated but high-cost medical screenings for diabetes and cardiovascular diseases. In this context, our findings presented here may have important implications in preventive public health policies in low-income Muslim Uyghur communities not only in China but worldwide.
